# The Tree Shrew as a Model for Cancer Research

**DOI:** 10.3389/fonc.2021.653236

**Published:** 2021-03-09

**Authors:** Tao Lu, Hongmei Peng, Liping Zhong, Pan Wu, Jian He, Zhiming Deng, Yong Huang

**Affiliations:** ^1^National Center for International Research of Bio-targeting Theranostics, Guangxi Key Laboratory of Bio-targeting Theranostics, Collaborative Innovation Center for Targeting Tumor Diagnosis and Therapy, Guangxi Talent Highland of Bio-targeting Theranostics, Guangxi Medical University, Nanning, China; ^2^Scientific Research and Education Department, The First People's Hospital of Changde City, Changde, China; ^3^The First People's Hospital of Changde City, Changde, China

**Keywords:** animal model, cancer, immunity, primate, tree shrew (*Tupaia belangeri*)

## Abstract

Animal disease models are necessary in medical research, and an appropriate animal model is of great importance for studies about the prevention or treatment of cancer. The most important thing in the selection of animal models is to consider the similarity between animals and humans. The tree shrew (*Tupaia belangeri*) is a squirrel-like mammal which placed in the order Scandentia. Whole-genome sequencing has revealed that tree shrews are extremely similar to primate and humans than to rodents, with many highly conserved genes, which makes the data from studies that use tree shrews as models more convincing and the research outcomes more easily translatable. In tumor research, tree shrews are often used as animal models for hepatic and mammary cancers. As research has progressed, other types of tree shrew tumor models have been developed and exhibit clinical manifestations similar to those of humans. Combining the advantages of both rodents and primates, the tree shrew is expected to be the most powerful animal model for studying tumors.

## Introduction

Cancer is the first or second leading cause of premature death globally and accounted for 4.5 million (29.8%) of the 15.2 million premature deaths from non-communicable diseases worldwide in 2016 (1). Cancer treatments include surgery, chemotherapy, radiotherapy, targeted therapy, etc. However, patients with advanced cancer often have multiple metastases, and surgery can no longer cure these patients. The complex tumor microenvironment increases the difficulty of cancer treatment. There is an urgent need to develop effective targeted therapies or immunotherapies for patients with advanced cancer. Mammalian models are of great importance in the investigation of the underlying mechanisms of cancer and in the development of treatments and interventions. Typically, the animals used as models need to be similar to humans in terms of anatomy, physiology, immunology and pathology and have the advantages of being easy to handle and inexpensive and requiring a short experimental cycle time. For example, mice are the most common mammalian model, and mice have the advantages of being inexpensive and easy to handle and requiring short experimental cycles; however, the immune system of mice is somewhat different from that of humans and is not suitable for all tumor studies (2).

A large number of animal models, such as *Drosophila* (3), mice (4), rabbits (5), dogs (6), pigs (7), rats (8), and rhesus macaque (9), are used in research to advance medicine. Each animal model has its strengths and weaknesses, and to date, no animal model has been created that can perfectly replicate human disease (10). For example, mice are widely used because of their clear genetic background, small size, and ease of handling. However, the genetic differences between mice and humans are substantial, making the clinical translation of many experimental achievements difficult. Large primates, such as orangutans, are very similar to humans in all respects and can very accurately simulate the physiopathological characteristics of human diseases. However, large primates are difficult to use as animal models due to high costs, operational difficulties, long experimental cycles and ethical restrictions (11). The tree shrew belongs to the family Tupaiidae, which is currently located in the order Scandentia. The genetic relationship between tree shrews and primates has been debated for a long time. The Kunming Institute of Zoology has recently reconstructed the phylogenetic relationships of tree shrews with 14 mammals (including six primate species) based on 2,117 single-copy genes in the whole genome of tree shrews, showing that tree shrews are more closely related to primates and resolving a long-standing controversy about the evolutionary status of tree shrews (12, 13). There are some common genetic properties between the tree shrew and human, such as genes/pathways involved in neuropsychiatric disorders and infectious diseases, common candidate drug targets squences, proteomic characterization of tree shrew liver and muscle tissues (14). There are also some unique genetic differences between the tree shrew and human. For example, the tree shrew lacked the *OPN1MW* [opsin 1 (cone pigments), medium-wave-sensitive] and *OPN1NW2* [(opsin 1 (cone pigments), medium-wave-sensitive 2] among the visually related human genes. The antiviral gene *RIG-I* (retinoic acid inducible gene I) was also lost during evolution (14). As a close relative of non-human primates, the whole genome of the tree shrew has been sequenced, and the results show that the nervous, immune and metabolic systems of the tree shrew are similar to those of humans (15–17). The relevant genomic data can be queried in the tree shrew database (TreeshrewDB) (http://www.treeshrewdb.org) (18). The tree shrew not only has the advantages of rodent models with small size, easy reproduction and short experimental periods but also has the advantage of high homology with primate models and humans. The data obtained from research using the tree shrew model are clinically instructive, and the tree shrew is well-suited for use as an animal model of human disease.

**Figure 1:**
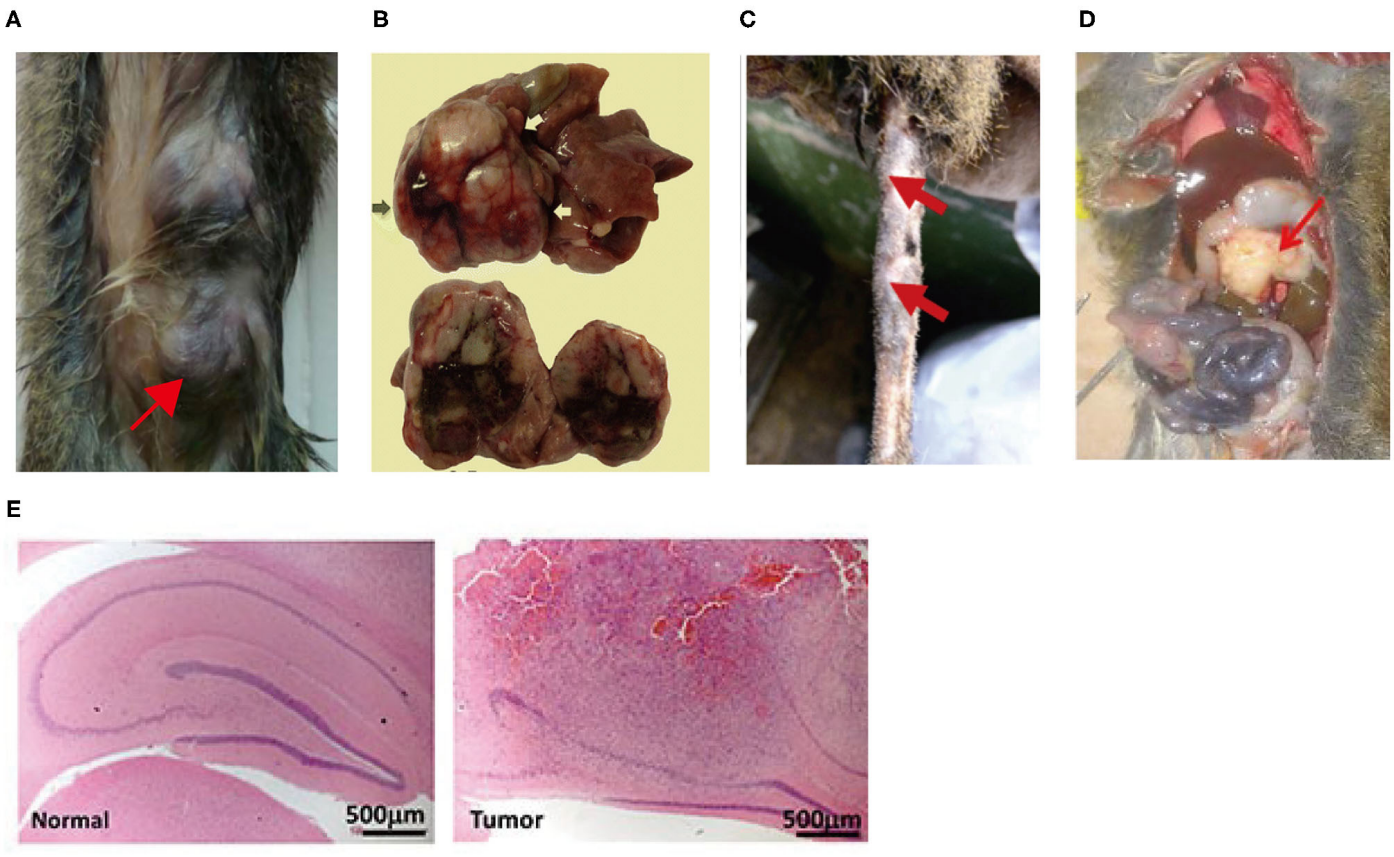
**(A)** Mammary tumors were induced in tree shrew by injection of lentivirus expressing the *PyMT* oncogene into mammary ducts (26); **(B)** Hepatocellular carcinoma was induced in tree shrew by inoculated with serum from HBV-infected patient or tree shrew (27). **(C)** A tree shrew basal cell carcinoma model was established by infecting tail skins with lentiviral SmoA1 (28). **(D)** Pancreatic ductal adenocarcinoma was induced in tree shrew by Injection of lentivirus expressing KRAS-shTp53-shCdkn2a/b into the pancreatic head of the tree shrew (29). **(E)** Malignant glioma was induced in tree shrew by engineering a lentiviral system for the transduction of mutant *H-RAS* and a shRNA against tree shrew *p53* (H&E) (30).

Tree shrews have been used in various disease models, such as infectious disease models (19), tumor models (20), metabolic disease models (21), and psychiatric and neurological disease models (22, 23). The development of inbred and transgenic tree shrews in recent years has further driven their widespread application (24, 25). Advanced tumors are complex and closely associated with immune, metabolic, and pathological phenotypes as well as genetic alterations. It is difficult for rodents to manifest disease characteristics that are similar in all respects to those of humans. The tree shrew has close homology with humans and exhibits pathological, physiological characteristics and immune responses similar to those of humans in tumor models, making tree shews become an appropriate animal model. However, the use of tree shrews for tumor modeling is still in the early stages, and some disadvantages remain, such as complex model construction methods and few tumor types; therefore, tree shrews are not widely used. In order to improve the efficiency of clinical translation of oncology research, it is imperative to promote the tree shrew tumor models. This paper reviews the anatomical features, physiological and biochemical characteristics, immune system and pathological tumor phenotypes of tree shrews, with emphasis on the various tree shrew tumor models constructed in recent years and their pathological phenotypes. Figure 1 shows tree shrew tumor models that have been successfully Induced in recent years.

## The Tree Shrew Anatomy

Compared to rodents, tree shrews, as an animal model, can effectively bridge the gap between basic research and translational medicine. The anatomy of the tree shrew is both similar to and different from that of other primates. The presence, abundance and distribution of neural stem and progenitor cells in tree shrews are more similar to those of primates than to those of rodents. The tree shrew is a novel model organism for studying human brain disease (31). Micro-computed tomography and microdissection show that the cochlear structure and cochlear rotations of the tree shrew are highly similar to those of humans, and thus, tree shrews can be used for research on ear diseases (32). The lungs of the tree shrew have three left lobes and four right lobes, whereas the lungs of humans have only two left lobes and three right lobes. The livers of both tree shrews and humans have left, middle, right and caudate lobes, and the difference is that in tree shrews, the gallbladder is in the central lobe, whereas in humans, it is located below the right lobe of the liver (33). Compared to humans, the tree shrew has a relatively simple intestinal structure but a small primitive caecum (34). In addition, the morphology and histology of tree shrew skin is very similar to those of human skin. The dermis and subcutaneous tissues contain hair follicles, sebaceous glands, sweat glands and acinar glands, where keratin 5 (KRT5) and keratin 10 (KRT10) are expressed in the cytoplasm of the skin. Thus, tree shrews are also suitable for elucidating the molecular mechanisms of various skin diseases (35).

## Tree Shrew Physiology and Biochemistry

Animal experiments are performed on the basis of characteristics that animals share with humans to some degree. In terms of physiology, it has been shown that the morphological characteristics of tree shrew blood cells are broadly similar to those of normal human blood cells. The red blood cell count (8.19 × 10^12^/L), red blood cell pressure volume (70.75%) and platelet count (450.17 × 10^12^/L) are all slightly higher than the normative reference values, probably due to the tree shrew's own highly active behavior. The values for leukocytes (2.21 × 10^9^/L) and neutrophils (0.85 × 10^9^/L) are below the lower limit of the human reference value. The lymphocytes, eosinophils, basophils and monocytes of tree shrews are generally within the reference range for humans (36–38). Biochemical analysis showed that only the creatinine results for tree shrews are below the lower limit of the human reference value, while the alanine aminotransferase and aspartate transaminase values are above the upper limit of the reference value. Urea, total protein, globulin, albumin, γ-glutamyl transferase, C-reactive protein and rheumatoid factor are all within the reference range for humans (36). In terms of metabolism, compared to rodents, tree shrews prefer a 5% concentration of sucrose but metabolize carbohydrates more poorly, which may account for the development of spontaneous diabetes in tree shrews (39). Moreover, the main stress-related hormone in tree shrews is cortisol, which is similar to humans, while in rats, it is corticosterone, which also suggests that tree shrews are good models for stress-related human diseases (40).

## Tree Shrew Immune System

### Tree Shrew Humoral Immunity

According to genomic comparisons, tree shrews are more similar to primates than to rodents in terms of immunity. The immune system of the tree shrew is conserved in many ways and can produce an immune response similar to that of humans, but in some ways, the immune response is also species-specific. Based on analysis of the structural domains and transcriptome of tree shrew immunoglobulins, tree shrews encode four classes of antibodies in addition to IgD; among these classes, IgM has the highest homology with that in primates (41). In terms of humoral immune responses, tree shrews and mice are very different. Infection with HSV-1 (Herpes simplex virus-1) produces systemic adverse reactions in mice but causes only minor infections in tree shrews (42). Similar to humans, tree shrews produce high levels of specific IgG antibodies against HSV-1, while mice produce relatively low levels of specific IgG antibodies. Similarly, the podoconjugate vaccine is successful in inducing bactericidal antibodies in tree shrews but not in mice (41). The immune behavior of tree shrews is more similar to that of humans, and tree shrews may be an important complement to mice for studying immunity.

### Tree Shrew Major Histocompatibility Complex (MHC)

MHCs are important for acquired immunity in vertebrates. It is well-known that MHC class I molecules are distributed on the surface of almost all nucleated cells. Acquired immune responses occur through the recognition and presentation of exogenous antigenic peptides to cytotoxic T lymphocytes (43, 44). The MHC class I molecule of the tree shrew consists of 357 amino acids, including a pilot peptide, α1 and α2 structural domains, an α3 structural domain, a transmembrane region and a cytoplasmic structural domain. Among these polymorphs, cysteine, CD8+ interaction and N-glycosylation sites are well-conserved and similar to those of humans (45). The MHC class II region of the tree shrew contains homologs of all the human class II molecules, including the classical class II genes *HLA-DP, HLA-DQ*, and *HLA-DR* and the non-classical class II genes *HLA-DM* and *HLA-DO* (15). The MHC class III gene in tree shrews is the most highly conserved compared to that in humans, differing only in the C4 region (15).

### Tree Shrew Pattern Recognition Receptors (PRRs)

Upon infection with exogenous pathogenic microorganisms, PRRs can rapidly respond and lead to the production of cytokines, such as interferon (IFN), inflammatory factors and complement proteins (46). These PRRs include Toll-like receptors (TLRs), NOD-like receptors (NLRs), RIG-like receptors (RLRs), and cytoplasmic DNA receptors (47). It has been found that tree shrews have 13 TLRs (including homologs of the mammalian tTLR1-tTLR9, tTLR11-tTLR13, and tTLR10 pseudogenes). Of these, tTLR8 and tTLR9 exhibit positive selection signals, which may be associated with the adaptive immunity triggered by pathogens. The mRNA transcriptional alterations of tTLR2, tTLR3, tTLR4, and tTLR8 in tree shrews infected with hepatitis C virus are consistent with the pattern observed in human hepatocytes (48). The RLRs consist of three members, namely, RIG-I, MDA5 (melanoma differentiation factor five) and LGP2 (laboratory of genetics and physiology two). RIG-I was found to be lost during the evolution of tree shrews, while MDA5 and LGP2 showed strong positive selection and induce type I IFN production. Despite the loss of RIG-I, the positively selected MDA5 locus in the tree shrew can replace RIG-I in its corresponding function (49).

### Tree Shrew Cytokines

IFNs play an important role in the immune system and are essential in infectious and inflammatory diseases, autoimmune diseases and cancer, in addition to their antiviral and antibacterial effects (50). Based on the available genome-wide data of tree shrews, Li et al. (50) identified type I IFN molecules (α (five subtypes), β, ω, κ, ε, and δ), type II IFN molecules (γ), and type III IFN molecules (λ1 and λ2/3) by large fragment nucleic acid sequence alignment and gene prediction; of these IFN molecules, IFNα and IFNβ in tree shrews are very similar to those in humans. The structure of the tree shrew type III IFN-λ3 and its receptors, IFNλR1 and IL10R2 (Interleukins 10 receptor 2) are also highly similar to their human counterparts (51). The structural features of type III IFN-λ3 and its receptors, IFNλR1 and IL10R2, in tree shrews are also highly similar to their human counterparts (51).

Chemokines are a family of small molecule proteins secreted by cells, and most chemokines mediate the migration of immune cells to the site of inflammatory infection, exerting anti-infective effects and modulating the immune microenvironment of tumors (52). The chemokines of the tree shrew are poorly understood, but some studies have shown that CXCL8 and its cognate receptors in tree shrews are structurally and functionally similar to and can cross react with those in primates. Human CXCL8 can bind to tree shrew CXCR1/2 to induce tree shrew PBMC migration, while overexpression of tree shrew CXCR1 in human HEK-293T cells can also promote cell migration *in vitro* (53). The same conclusion was also confirmed with CXCL12. Tree shrew CXCL12 and its ligand, CXCR4, are highly similar in structure to their human homologs, and the recombinant human CXCL12 protein can directly mediate the migration of tree shrew lymphocytes *in vitro* (54).

Interleukins (ILs) are cytokines that play an important role in inflammatory responses by activating and regulating immune cells and mediating T and B cell activation, proliferation and differentiation. Most ILs also play an essential role in tumor immunity (55–57). Immunological factors are vital indicators for the evaluation of tree shrews as models of human disease. Tree shrew interleukin 2 (tIL-2) is a 154-amino acid protein with 80% amino acid sequence homology to human IL-2. Tree shrew IL-2 has one more N-glycosylation site, and the rest of the overall structure is essentially similar to that of human IL-2 (58). Interleukin 6 (IL-6) is also important in the progression of infection, inflammation and cancer. The full length of the tree shrew IL-6 (tIL-6) gene is 5265bp, with five exons and four introns. tIL-6 has a 25-amino acid signal peptide and a conserved structural domain, which is consistent with the protein profile of human IL-6 (59). In terms of amino acid sequence, the homology of human IL-6 with chimpanzee, monkey, tree shrew, rat and mouse IL-6 is 98.11, 96.70, 52.36, 40.65, and 30.05%, respectively (59). A phylogenetic analysis based on amino acid sequences showed that interleukin 7 (IL-7) in tree shrews differs considerably from that in humans. The splice regions of the tree shrew IL7-sv2, IL7-sv4, and IL7-sv5 transcripts are similar to those of the human IL7d5, IL7d3/4, and IL7d3/4/5 transcripts, respectively. However, the IL7-sv1, IL7-sv3, IL7-sv6, IL7-sv7, IL7-sv8, IL7-sv9, and IL7-sv10 transcripts of the tree shrew have no homology in humans (60). Interleukin 21 (IL-21), an immunomodulatory cytokine produced by natural killer (NK) cells and T cells, is pleiotropic in immune and non-immune cells and mediates the anti-tumor immune effects of T and NK cells (61, 62). The nucleotide sequence homology between tree shrew IL-21 (tIL-21) and human IL-21 is 83.33%, and the amino acid sequence homology is 69.93%. The secondary structure, hydrophobicity and surface charge distribution of tIL-21 are also similar to those of IL-21 in humans (63). In addition, tIL-21 can bind to human IL-21 antibodies, and recombinant human IL-21 can also stimulate changes in the splenic lymphocyte profile of tree shrews. In other words, tIL-21 is immune cross-reactive with human IL-21 (63).

## Tree Shrew Pathological Phenotype of Spontaneous Tumors

Tree shrews can spontaneously develop tumors. Since 1966, when Elliot et al. (64) reported a case of spontaneous breast cancer in a tree shrew. Spontaneous tumors, including hepatocellular carcinoma (65), genital tumors (66), Jugulo-sternal-gland tumors (67), lung cancer (68), malignant lymphoma, skin cancer, gastrointestinal tract tumors, etc., (69) have been reported in tree shrews. It has been found that spontaneous mammary carcinomas in tree shrews have a high genetic homology with human mammary carcinomas, and some cases have familial mammary tumor characteristics. The spontaneous mammary tumors in tree shrews can be histochemically classified into tubulopapillary carcinoma and intraductal papillary adenoma. Of these tumors, 100% are positive for progesterone receptor (PR), 91.3% are positive for estrogen receptor (ER) and 4.3% are positive for human epidermal growth factor receptor 2 (HER-2), according to immunohistochemical analysis (70). The *PTEN/PIK3CA* gene is frequently mutated in spontaneous mammary carcinoma in tree shrews, whereas this phenomenon has not been observed in mouse mammary carcinoma models; thus, tree shrews may be a promising animal model for this type of mammary carcinoma (71). Eight spontaneous lung tumors (four fine bronchial tubular adenomas, two fine bronchial adenocarcinomas and two squamous cell carcinomas) were observed in 54 adult tree shrews at the German Primate Center between 1978 and 1994 (68). The similarity of the histopathological phenotype of tree shrew tumors to that of human tumors provides strong evidence for the use of tree shrews as cancer models.

## Methods for Constructing Tree Shrew Cancer Models and Their Pathological Phenotypes

### Hepatocellular Carcinoma Model

Hepatocellular carcinoma is one of the most prevalent cancers in the world, and it is also the second leading cause of cancer-related death (72). To date, there has been no effective strategy for advanced hepatocellular carcinoma. Research with animal models can help increase our knowledge of hepatocellular carcinoma and improve the treatment strategies (73). As early as the 1880s Reddy et al. (20) induced hepatocellular carcinoma by feeding tree shrews highly pure aflatoxin B1 (AFB1). Six of the female tree shrews (100%) and three of the six male tree shrews (50%) developed hepatocellular carcinoma. The consumption of AFB1 by the tree shrews that developed hepatoma was 24–66 mg. It was later discovered that, unlike rodents, tree shrews are susceptible to hepatitis B virus (HBV) to a similar extent as humans and exhibit the same symptoms. This method induced a model of hepatocellular carcinoma in tree shrews that was more like human HBV hepatocellular carcinoma (27, 74). After feeding AFB1 to tree shrews infected with hepatitis B virus (200–400 μg/kg body weight, once a day, six times a week), the incidence of primary hepatoma was found to be higher in the tree shrews exposed to AFB1 (52.94%) than in those infected with HBV alone (11.11%) or exposed to AFB1 alone (12.50%) (75). Moreover, the consumption of AFB1 by the tree shrews that developed hepatoma was much lower (15-16 mg) than the dose used in Reddy's study. These results demonstrate a synergistic effect of HBV and AFB1 in the development of primary hepatocellular carcinoma. Later, it was also found that the hepatocellular carcinogenesis induced by AFB1 in tree shrews was significantly inhibited by oltipraz, and the gene expression profile of hepatocellular carcinoma induced by different factors was also different (76, 77). Differential gene expression was predominantly upregulated in the AFB1 group and downregulated in the AFB1+HBV group. This difference helps identify genes that play a key role in hepatocellular carcinogenesis. In addition, CuZn-superoxide dismutase (SOD1) and glutathione S-transferase A1 (GSTA1) were found to be downregulated in both tree shrew and human hepatocellular carcinoma tissues, and the downregulation of these proteins may have an important link to the development of hepatocellular carcinoma. As in humans, the oncogene *p53* was mutated in the tree shrew model of hepatocellular carcinoma. Upregulation of the p21 protein and oncogene *RAS* was observed in the pre-hepatocarcinogenesis stage, suggesting that p21 protein may be an early marker of hepatocellular carcinoma (78, 79).

### Mammary Cancer Model

Mammary cancer is the most common cancer among women, and it is also the second leading cause of cancer-related death among women (80). 7, 12-dimethylbenz[a] anthracene (DMBA) is considered an effective chemical for inducing breast cancer and has been used to induce breast cancer in rodents (81, 82). After a study of primary mammary cancer in tree shrews, Xia et al. (71) selected female tree shrews aged 3–4 months in which to induce mammary cancer by the oral administration of 20 mg DMBA every 3 weeks for a total of three times. Fifteen of these tree shrews were implanted with medroxyprogesterone acetate (MPA) pellets after 9 weeks. The results showed that the mammary cancer incidence was only 12% among the tree shrews treated with DMBA alone, while the combined use of DMBA and MPA resulted in an incidence of 50%. The PTEN/PIK3C mutation was the most common mutation observed in this model. The pathological phenotypes of tree shrew mammary cancer induced by the combination of DMBA and MPA showed that these tumors were positive for ERα, PR and cytokeratin 5/6 (CK5/6) and negative for Her-2 (83). Ge et al. (26) injected a lentivirus expressing the oncogene *PyMT* into the mammary ducts of tree shrews to induce mammary cancer, and this approach overcame limitations of low induction efficiency and long latency period associated with the DMBA method. This method induces mammary cancer with a short latency period (about 3 weeks) and a high tumor incidence (100%). The majority of the induced tumors are papillary carcinomas with the following characteristics: ER (+), PR (+), Ki-67 (+) and Her-2 (–). *Cisplatin* and *Epidoxorubicin* significantly inhibit the progression of *PyMT*-induced mammary tumors (26).

### Lung Cancer Model

Lung cancer is the leading cause of cancer-related deaths worldwide, and in 2017, more deaths occurred from lung cancer than from breast, colorectal, prostate and brain cancers combined (1). In 1980 Rao et al. (84) studied the carcinogenic effects of 2,2'-dihydroxy-di-n-propylnitrosamine using tree shrews as experimental subjects. These tree shrews were subcutaneously injected with a dose of 250 mg/kg body weight weekly for 80 weeks. Up to 102 weeks, 78% of the female and 89% of the male tree shrews were observed to have developed pulmonary adenomas, including two males with bronchoalveolar carcinoma, and Clara cells were a major component of these tumors. Nine percent of the tree shrews also had squamous cell carcinoma of the skin and hepatocellular carcinoma. This was the first time a tree shrew lung cancer model was constructed. Bronchial epithelial hyperplasia was later induced by Chen et al. (85) using Xuanwei bituminous coal dust PM10, but the animals died within a week. Ye et al. (86) attempted to create a lung cancer model by dripping iodinated oil suspensions of 3-methylcholanthrene and diethylnitrosamine at different concentrations into the trachea of tree shrews, but unfortunately, all of the animals in the experimental group died. Although the construction of a tree shrew lung cancer model has not always been easy and has even been difficult, Che's recent successful induction of a tree shrew model of pulmonary fibrosis using *bleomycin* is good news (87).

### Basal Cell Carcinoma Model

The skin anatomy of tree shrews is very similar to that of humans and is very suitable for use as a model of skin diseases to elucidate the underlying molecular mechanisms (35). Basal cell carcinoma (BCC) is the most commonly occurring non-melanoma skin cancer (88). Studies have shown that the main oncogenic mutations in BCC are mutations in *ptch1* and *Smo*, and these mutations cause abnormal activation of the Hedgehog signaling pathway (89, 90). Jiang et al. (28) injected the lentivirus pCDH-mSmoA1 (5.6 × 10^5^ TU), which expresses the *SmoA1* gene, and shRNA lentivirus *shp53* (2 × 10^5^ TU), which targets the tree shrew *p53* gene, into the back and tail of tree shrews. The tree shrews injected with SmoA1 alone had a BCC tumor formation rate of 40% after 2 weeks, and this rate increased to 60% after 6–8 weeks; however, the tree shrews injected with both SmoA1 and p53-shRNA had a tumor formation rate of 70% after 2 weeks, and this rate reached 100% after 4 weeks. RT-PCR (Reverse Transcription-Polymerase Chain Reaction) showed upregulated mRNA expression of the oncogenic genes *ptch1* and *Gli1*. The overexpression of SmoA1 successfully induced tree shrews BCC, and the downregulation of p53 accelerated the formation tree shrew BCC. The H&E (hematoxylin-eosin staining) results show that the skin basal cell carcinoma model constructed by this method exhibits pathological features similar to those of observed in humans, such as skin cell proliferation, hair follicle rupture, hyperpigmentation and nuclear explosion expansion.

### Glioblastoma Model

Glioblastoma is one of the most malignant tumors, with an average survival time of only 15 months, and no current treatment is effective in prolonging survival (91, 92). It is particularly vital to develop an animal model that can effectively simulate human glioblastoma to study the disease mechanisms and therapeutic strategies for treating it. In a study, the overexpression of *H-RAS* and silencing of the *p53* gene were found to be effective in inducing gliomas that were identified as mesenchymal glioblastomas, the subtype with the highest degree of malignancy (30). Tumor formation began after 4 weeks after injection of 4 μl of lentivirus expressing the mutant *H-RAS* gene and *shp53* into the hippocampal region of the tree shrew brain. Notably, tree shrew glioblastoma has features consistent with human glioblastoma, such as necrosis, microvascular proliferation and pseudopolyps. The tree shrew glioblastoma model provides researchers with a more accurate animal model.

### Pancreatic Cancer Models

Pancreatic ductal carcinoma (PDAC) accounts for 90% of human pancreatic cancers. Many studies have indicated that acinar cells are the cells of origin of PDAC (93–95). *KRAS*^*G*12*D*^ can induce pancreatic cancer in embryonic mice but not in adult mice (96). Tu et al. (29) found that lentiviruses can specifically infect the acinar cells of the adult tree shrew pancreas. By injecting 1 × 10^7^ lentiviral particles expressing KRAS-shTp53-shCdkn2a/b (total of 20 μl) into the pancreatic head of tree shrews *in situ*, a completely exposed pancreatic cancer model was induced after 3–7 weeks, whereas all the other injected control lentiviral particles failed to produce tumors. The upregulation of the oncogene *KRAS* and the loss of function of the tumor suppressor genes *TP53, CDKN2a*, and *CDKN2b* are thus necessary for the induction of pancreatic cancer. This tumor model involves an intermediate differentiated ductal adenocarcinoma that expresses human pancreatic cancer markers, such as keratin 19 (CK19), mucin 5 (Muc5), matrix metallopeptidase 7 (MMP7), and hes family bHLH transcription factor 1 (Hes1). The biological features of human PDAC are reproduced in this tree shrew model of pancreatic cancer, which is of great value for basic research on pancreatic cancer.

The tree shrew model of carcinoma induction *in situ* is more similar in all respects to the manifestations of human disease than the traditional rodent model of transplanted tumors, and thus, the tree shrew model has more research value. The methods of construction the tumor models described above, as well as their characteristics, are summarized in Table 1.

**Table 1:** Tree shrew cancer models.

**Cancer models**	**Methods**	**Characteristics**	**References**
Hepatocellular carcinoma (HCC)	Intermittent administration of 2 mg/kg of high purity aflatoxin B1 in the diet For 172 weeks	9/12 of the tree shrews developed liver tumors between 74 and 172 weeks of the experiment. All nine tree shrews were well to poorly differentiated hepatocellular Carcinomas.	(20)
	Tree shrews infected with human HBV are fed AFB1 (200–400 μg/kg body weight per day) for a total of 15-16 mg 6 days a week	Within 83–137 weeks, the incidence of HCC in tree shrews infected with HBV and exposed to AFB1 (52.94%) was significantly higher than that in groups infected with HBV (12.5%) or only exposed to AFB1 (11.11%). Thirteen cases of liver tumors were all hepatocellular carcinoma, including eight cases of trabecular type, three of adenoid type, and two of poorly-differentiated type. The tumors were single or multi-nodular.	(75)
Mammary cancer	30 tree shrews were administered intragastrically with 1 ml peanut oil containing 20 mg DMBA, once every 3 weeks for a total of 3 times. After 9 weeks, 15 of them were implanted with 150 mg MPA.	The combined administration of DMBA + MPA has a tumor formation rate of 50%, Immunohistochemistry: ER (+), PR (+), CK5/6 (+), Her-2 (–). *PTEN* and *PIK3C* genes are frequently mutated in this breast cancer model	(71)
	10 ul of lentivirus overexpressing the *PyMT* cancer gene was injected into the mammary ducts of the tree shrews.	The incubation period was 3 weeks, after 7 weeks all tree shrews developed mammary tumors, mainly papillary carcinomas. Immunohistochemistry: ER (+), PR (+), Her-2 (–).	(26)
Lung cancer	DHPH was injected subcutaneously once a week at 250 mg/kg body weight for 80 weeks.	Up to 102 weeks 78% of female and 89% of male tree shrews were observed to have developed lung adenomas, with Clara cells being a major component of these tumors, and two of these were bronchoalveolar carcinomas. A further 9% of the tree shrews had squamous cell carcinoma of the skin and hepatocellular carcinoma.	(84)
Basal cell carcinoma	The lentivirus expressing the oncogene SmoA1 (5.6 × 10^5^ TU) and the shp53 (2 × 10^5^ TU) lentivirus were injected into the skin of the back and tail of the tree shrew, with a total volume of 10 μl.	The combined injection of SmoA1 and p53-shRNA virus group showed a BCC-like phenotype of 70% after 2 weeks, and the tumor formation rate reached 100% after 4 weeks. *Ptch1* and *Gli1* mRNA expressions were up-regulated.	(28)
Glioblastoma (GBM)	4 μl lentiviruses (3 × 10^11^/ml titered by real-time PCR) expressing H-RAS and shp53 were injected into the hippocampus of the tree shrew.	*DH1, TERT, EGFR, PTEN, ATRX* and other key factors are expressed in gliomas as in human GBM, and *EGFR* is upregulated in tree shrew GBM.	(30)
Pancreatic cancer	Injection of 20 ul lentivirus expressing KRAS-shTp53-shCdkn2a/b into the pancreatic head of the tree shrew	The tumor formed after 3–7 weeks, which was a moderately differentiated ductal adenocarcinoma. Immunohistochemistry: CK19 (+), Muc (+), MMP7 (+), Hes1 (+).	(29)

*TU: transducing units; (+): positive; (–): negative*.

### Summary and Prospects

In summary, compared to rodents, tree shrews are more similar to humans in terms of genetics, immune system and nervous system. In addition, tree shrews have more advantages than large primates in terms of breeding, domestication, handling and ethics. As an emerging animal model of human disease, the tree shrew has unique advantages for the study of viral infections (97), depression (98) and myopia (99). The advantages of the tree shrew primary tumor model are also quite prominent in antitumour research. In addition to the physiological, biochemical and pathological similarities between tree shrews and humans, the mechanisms of the immune response in tree shrews are also highly conserved with those in humans (53). Analysis of drug targets and predictions at the genomic and transcriptional level in tree shrews revealed 3,482 proteins predicted as drug targets, including homologs for cancer chemotherapy, depression, and cardiovascular disease. More than half of these target proteins have higher similarity to human target proteins than those in mice (100). In other words, the tree shrew model is of greater clinical value in terms of research about tumor therapies, particularly immunotherapies. However, there are still relatively few types of tree shrew tumor models because little research has been done on tree shrews. A well-developed method for constructing tree shrew tumor models is one of the keys to the extension of tree shrew applications. Methods for constructing tree shrew tumor models are still being explored, and the existing methods still have some drawbacks, such as long construction time, difficult operation, low success rate and difficulty in standardization. Mechanistic studies at the cellular and small molecule levels in tree shrews are of great importance for improving the modeling schemes. Based on the current study, the approach of using lentivirus transduction into cells *in situ* to overexpress oncogenes or inhibit tumor suppressor gene expression seems to be more effective for the construction of tree shrew tumor models, while the approach of drug induction appears to require longer durations and have a lower success rate.

Fortunately, the Kunming Institute of Zoology has achieved standardized captive domestication of tree shrews and can provide more than 1,000 experimental tree shrews each year (11). However, there are currently some problems with the use of tree shrews as tumor models. The greatest problem is that there are currently no inbred tree shrew strains with clear genetic backgrounds. The large individual differences between tree shrews are one of the factors that make it difficult to standardize experiments. Fortunately, scientists at CAS have now started to work on establishing tree shrew strains. There is still a long way to go in generating inbred strains of tree shrews, but this is a crucial key step for standardizing experimental tree shrews. In addition, supporting tools for tree shrews research have not yet been developed. For example, tree shrew cell lines, tree shrew-specific antibodies, corresponding assays, etc., must be developed. However, the homology between tree shrews and humans makes some of the immunizations cross-reactive, and thus, in many cases, human cell lines and antibodies can be used. There is no doubt that the tree shrew is becoming increasingly important in the field of medical research, and the results achieved are considerable. Further study of the tree shrew's immune system and its physiological and biochemical characteristics may provide more insight into the development of tumor models. It is believed that in the near future, tree shrews could replace large primates as the most powerful tool for cancer-related pre-clinical research.

## Author Contributions

YH and ZD contributed to the conception and design of the review. TL and HP wrote the manuscript. LZ, PW, and JH revised the manuscript. All authors contributed to manuscript revision, read, and approved the submitted version.

## Conflict of Interest

The authors declare that the research was conducted in the absence of any commercial or financial relationships that could be construed as a potential conflict of interest.
